# The Efficacy and Safety of Norepinephrine and Its Feasibility as a Replacement for Phenylephrine to Manage Maternal Hypotension during Elective Cesarean Delivery under Spinal Anesthesia

**DOI:** 10.1155/2018/1869189

**Published:** 2018-12-31

**Authors:** Xian Wang, Xiaofeng Shen, Shijiang Liu, Jianjun Yang, Shiqin Xu

**Affiliations:** ^1^Department of Anesthesiology, Obstetrics and Gynecology Hospital Affiliated to Nanjing Medical University, Nanjing, China; ^2^Department of Anesthesiology, The First Affiliated Hospital of Nanjing Medical University, Nanjing, China; ^3^Nanjing Medical University, Nanjing, China

## Abstract

Maternal hypotension commonly occurs during spinal anesthesia for cesarean delivery, with a decrease of systemic vascular resistance recognized as a significant contributor. Accordingly, counteracting this effect with a vasopressor that constricts arterial vessels is appropriate, and the pure *α*-adrenergic receptor agonist phenylephrine is the current gold standard for treatment. However, phenylephrine is associated with dose-dependent reflex bradycardia and decreased cardiac output, which can endanger the mother and fetus in certain circumstances. In recent years, the older, traditional vasopressor norepinephrine has attracted increasing attention owing to its mild *β*-adrenergic effects in addition to its *α*-adrenergic effects. We search available literature for papers directly related to norepinephrine application in spinal anesthesia for elective cesarean delivery. Nine reports were found for norepinephrine use either alone or compared to phenylephrine. Results show that norepinephrine efficacy in rescuing maternal hypotension is similar to that of phenylephrine without obvious maternal or neonatal adverse outcomes, and with a lower incidence of bradycardia and greater cardiac output. In addition, either computer-controlled closed loop feedback infusion or manually-controlled variable-rate infusion of norepinephrine provides more precise blood pressure management than equipotent phenylephrine infusion or norepinephrine bolus. Thus, based on the limited available literature, norepinephrine appears to be a promising alternative to phenylephrine; however, before routine application begins, more favorable high-quality studies are warranted.

## 1. Introduction

Maternal hypotension is a physiological response during cesarean delivery with spinal anesthesia that significantly contributes to adverse maternal outcomes such as nausea, vomiting, dizziness, and even cardiovascular collapse. In addition, compromised placental perfusion raises concerns of fetal acidosis, hypoxia, and even postnatal neurological injury. Thus, effective prevention and treatment of maternal spinal hypotension is of great clinical significance.

At present, phenylephrine is the first-line vasopressor used in obstetric anesthesia to manage maternal spinal hypotension. However, in recent years, another vasopressor norepinephrine has attracted increasing attention, as a feasible substitute for phenylephrine in obstetric anesthesia. Use of norepinephrine to prevent or treat maternal spinal hypotension during obstetric anesthesia is a recent advance and thus available data are limited. Further, concerns exist regarding the use of such potent vasopressor in a nonintensive setting, such as the operating room [1]. In this paper, we search for recently published studies using post-spinal norepinephrine application. We then summarize norepinephrine efficacy and safety in managing maternal hypotension during cesarean section with spinal anesthesia. Finally, we discuss its feasibility as a substitute for the current gold standard phenylephrine that is used in this context.

## 2. Materials and Methods

### 2.1. Information Retrieval

**Figure 1:**
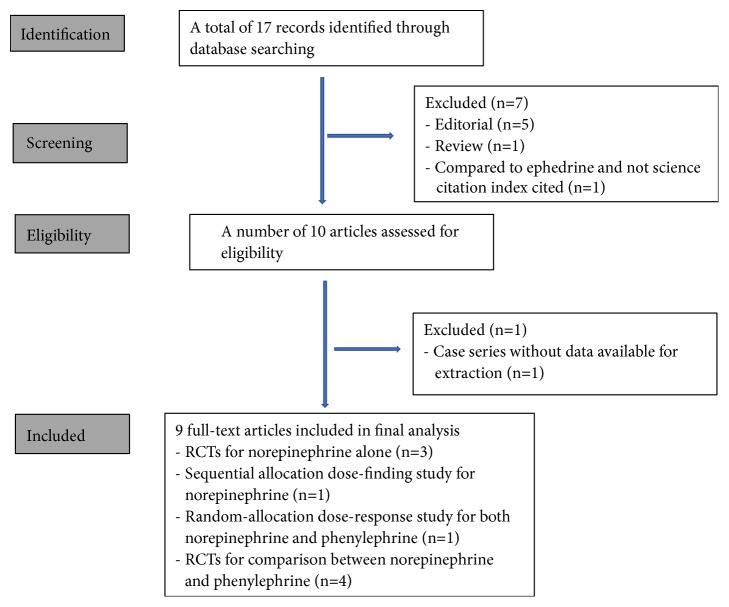
Flowchart of studies included. Footnote: The excluded two articles that neither editorial nor review are as follows: Selim MF. Norepinephrine versus ephedrine to maintain arterial blood pressure during spinal anesthesia for cesarean delivery: a prospective double-blinded trial. Anesthesia, essays, and researches 2018; 12: 92-97. Ngan Kee WD. Norepinephrine for maintaining blood pressure during spinal anaesthesia for caesarean section: a 12-month review of individual use. Int J Obstet Anesth 2017; 30: 73-74.

We performed a systematic literature search in the database websites of Pubmed, Embase, and GeenMedical to find reports directly related to postspinal norepinephrine application using the keyword phrase ‘norepinephrine AND obstetric anesthesia' and set the search end point at Oct 31, 2018. Reference lists of these retrieved papers and reviews related to vasopressor use during spinal anesthesia were also screened to minimize possible omissions. Eliminating editorial, review, and nonscience index cited reports, we found nine suitable reports (details shown in Table 1). A detailed flowchart of literature retrieval is shown in Figure 1.

### 2.2. Assessment of Articles

All nine included reports were published from 2015 to 2018. Three are randomized controlled trials (RCTs) for norepinephrine (NE) alone [2–4], one is a sequential allocation dose-finding study for NE [5], one is a random-allocation dose-response study for both NE and phenylephrine (PE) [6], and the remaining four papers are RCTs that compare these two vasopressors [7–10]. All enrolled subjects were healthy parturients without comorbidities that had an intrathecal injection of bupivacaine or ropivacaine in combination with opioid fentanyl and/or morphine. Similarly, fluid loading and the left tilt supine position were used for all parturients in all reports. Noninvasive hemodynamics were monitored in four reports using USCOM [3, 9], LIDCO [4] or Nexfin [7]. Of note, results in Ngan Kee et al. 2017 [8] are a reanalysis of data from their study in 2015 [9]; the first paper focuses on hemodynamic stability and the second on cardiac output (CO). As a result, there are actually three groups of RCT trials available that compare NE and PE. This small sample size, along with the heterogeneity in primary observational outcomes and administration regimens, does not provide enough statistical power to perform a meta-analysis. Thus, we only descriptively present the results of the nine reports and discuss potential advantages of NE compared to PE.

## 3. Results

### 3.1. Efficacy Evaluation of NE for Obstetric Parturients

Efficacy to rescue maternal hypotension is of main concern with regard to vasopressor choice and NE efficacy evaluated in these reports is detailed in Table 2. In 2015, Ngan Kee et al. [9] compared parturients undergoing elective cesarean delivery with spinal anesthesia receiving a variable rate continuous infusion of NE 0-5 *μ*g/min to those receiving PE 0-100 *μ*g/min, with a computer-assisted closed loop feedback algorithm for maintaining systolic blood pressure (SBP) near baseline. Both groups had similar incidences of hypotension, hypertension, maternal side effects, and neonatal outcomes. However, NE group had a lower incidence of bradycardia and a greater CO. The authors suggested the observed CO with NE is primarily related to a greater heart rate (HR) that possibly comes from its weak *β*-adrenergic agonist property, which is absent in PE. A reanalysis of these data further showed that NE infusion was associated with a more precise control of SBP compared to PE infusion, as demonstrated by a decreased MDPE (the median of all values of performance error for each patient), MDAPE (the median of the absolute values of performance error for each patient), and Wobble (the median value of the differences between each value of performance error and MDPE) [8]. Such superiority of NE is plausibly attributable to its pharmacological properties including a fast onset and short duration [11] that make accurate titration possible. However, computer-assisted infusion technology is currently not recommended for clinical practice [1]. Thus, Ngan Kee et al. explored the efficacy of a simpler algorithm, using manually controlled variable rate infusion of NE 0-5 *μ*g/min to maintain SBP near baseline, compared to using a rescue bolus of 5 *μ*g whenever hypotension occurred [3]. Compared to the rescue bolus, the manually controlled infusion regimen was associated with a lower incidence of hypotension, a similar CO, and a better control for SBP stability. Although a relatively larger dose of NE was applied, no maternal or neonatal adverse outcomes were observed. Considering intravenous bolus is still the favored medication paradigm for most anesthesiologists [12], a final RCT trial compared the efficacy of equipotent bolus NE and PE for maternal hypotension with the incidence of bradycardia as primary outcome. Compared to PE, an NE intermittent bolus resulted in a nearly 71% reduction in the occurrence of bradycardia, which indicates its potential for better maintenance of CO [10].

A lower MDPE indicates a lower level of SBP for NE, suggesting 0-5 *μ*g/min of NE might be less potent (not equivalent, as expected) than PE 0-100 *μ*g/min [8]. A subsequent dose-response study for rescuing the first episode of maternal hypotension calculated an ED90 of 18 *μ*g versus 239 *μ*g for NE and PE, respectively in obstetric anesthesia, a potency ratio of approximately 13:1 [6]. Another dose-finding study found an ED90 of NE for SBP maintenance to higher than baseline value was approximately 6 *μ*g [5]; the majority of those receiving a dose lower than 6 *μ*g still presented with hypotension (5 of 6 cases). Difference in the timing of intervention, at SBP < 80% baseline in Ngan Kee et al. [6] and at SBP < baseline in Onwochei et al. [5], might account for the dosing differences observed in these two studies.

Three RCT trials explored NE efficacy for SBP maintenance using a fixed rate infusion regimen [4, 7]. In Chen D et al. [4], NE 5 or 10 *μ*g/kg/h, a dose equal to 5 or 10 *μ*g/min for a parturient with a weight of 60 kg, provided a better BP maintenance without increased incidence of hypertension compared to a dose of 15 *μ*g/kg/h. Vallejo et al. [7] compared NE 0.05 *μ*g/kg/min and PE 0.1 *μ*g/kg/min, a dose that equivalent to NE 3 *μ*g/min and PE 6 *μ*g/min for a 60 kg parturient, and found the former had a less need for ephedrine. However, a similar high incidence of need for rescue bolus of both vasopressors (PE and ephedrine), 65.8% vs 48.8%, suggested such dosing might not be adequate to rescue maternal hypotension. Further, in a recently published trial [2], two doses, 0.05 or 0.075 *μ*g/kg/min dose of NE infusion, were both effective in reducing postspinal hypotension and NE 0.075 *μ*g/kg/min did not provide an additional advantage compared to 0.05 *μ*g/kg/min other than a slighter higher SBP (nearly 5 mmHg). Thus, the authors suggested that 0.05 *μ*g/kg/min, a dose equal to 3*μ*g/min for a 60 kg parturient, is the best infusion dose for NE during spinal anesthesia with cesarean delivery.

The ultimate goal of hemodynamic management is to maintain adequate maternal CO and uteroplacental perfusion. Four reports measured CO [3, 4, 7, 9] with various devices (USCOM, LIDCO, Nexfin), and only Ngan Kee et al. [9] reported a greater CO in the NE vs. PE group. However, it should be noted that, except for the first five minutes when CO in the PE group was at 94% baseline, CO was above preanesthetic baseline in both groups at all measured timepoints in the study. In addition, the subtly greater CO observed did not translate into clinical advantages, as no differences in maternal or neonatal outcomes were observed for NE compared to PE. This may be because all the enrolled parturients were healthy ones and without comorbidity. CO advantage should be further clarified in high risk situations such as maternal cardiac disease, placental insufficiency, or compromised fetal status.

### 3.2. Safety Evaluation of NE for Obstetric Parturients

Commonly used variables to evaluate vasopressor safety include maternal side effects such as nausea, vomiting, dizziness, shivering, and local tissue ischemia and neonatal outcomes including Apgar score and umbilical blood gas analysis. Although not used as primary observational outcomes, a majority of included reports measured these parameters (Table 3). For maternal outcomes, most studies found no differences in nausea or vomiting between NE and PE [2–5, 8–10]. A high incidence of emesis in Vallejo M et al. [7] was observed for PE compared to NE (26.3% vs 16.3%); however, a chi-square test easily showed that this difference was not statistically significant with a* P* = 0.29 rather than a value < 0.001, as provided by the authors. Thus, available literature consistently shows a maternal safety in terms of nausea or vomiting.

NE-induced vasoconstriction and skin necrosis is another concern for NE application in obstetric anesthesia, where a peripheral rather than central vein is commonly used [13]. Chen D et al. [4] observed skin color, an indicator of peripheral vascular constriction, and they found the incidence of pale skin was relatively low and similar among groups. In addition, Ngan Kee et al. [3, 6] and Onwochei et al. [5] suggested that NE is safe for local tissue perfusion since NE is diluted before use and administered in a running fluid for a relatively short duration, thus reducing the risk of tissue ischemia. Further, an equal potency of NE infusion or bolus has a theoretically similar vasoconstrictive potency as the currently used PE, so that risk should be no different to that posed by PE. Furthermore, a previous study showed spinal anesthesia increases skin perfusion and that this effect is not counteracted by NE application [14]. Collectively, results suggest NE likely has no adverse effect on local tissue perfusion in patients with spinal anesthesia for commonly used infusion or bolus doses. Commercially available NE (Levophed) does not specify that NE needs to be given centrally, indicating only that it should be via a large vein, preferably antecubital [15].

The safety profile of NE for the fetus and neonate is another consideration. Apgar scoring was performed in all reports and no obvious detrimental effects observed for NE. Similarly, UA or UV blood gas analysis showed no adverse effects for NE with regard to pH, PCO_2_, PO_2_, HCO_3_, and base excess (BE) values. Onwochei et al. [5] observed a 6 *μ*g dose of NE was related to a more negative BE compared to smaller doses, thus raising the concern for fetal acidosis of NE. However, such lower BE was still within a normal range and this study was not powered enough to detect a true difference in NE safety with different doses. NE infusion may increase maternal and neonatal glucose, partially resulting from an increase in catecholamine stimulated glucose metabolism increase and a *β*-receptor-mediated insulin decrease [4, 9]. NE is not expected to readily cross the placenta, which has the ability to break down catecholamines. In fact, as suggested by Ngan Kee et al. [9], the use of NE may actually reduce fetal catecholamine level compared to PE, eliminating the potential stimulation of fetal metabolism and acidemia often seen with ephedrine.

## 4. Discussion

Understanding of maternal hypotension postneuraxial anesthesia is improving as new evidence accumulates. As early as the 1940s, studies suggested that aortocaval compression impedes venous return and sympathetic block exacerbates venous blood pooling in the lower extremity to synergistically reduce venous return, decreasing CO and causing maternal hypotension [16, 17]. Thus, therapeutics such as fluid loading, left uterine displacement, left table tilt, or mechanical lower extremity compression have long been favored to expand intravascular volume based on such understanding [18, 19]. However, these strategies did not consistently show the expected efficacy in alleviating maternal hypotension.

Then, nearly a decade ago, studies suggested, the primary effect postspinal anesthesia is a decrease of systematic vascular resistance (SVR) secondary to small artery vasodilation[20], along with a modest degree of venous vasodilation [21, 22]. Since then, prevailing opinion suggests loss of arteriolar tone and a significant decrease of SVR is likely the main mechanism involved in maternal spinal hypotension. In this context, use of vasopressors, given their arterial vessels constriction property, is rational and recommended to rescue spinal anesthesia-induced hypotension.

At present, ephedrine, PE, and NE are three commonly used vasopressors during elective cesarean delivery with spinal anesthesia. Other alternatives exist, including methoxamine, mephentermine, and metaraminol, but these are not first choices for most clinicians due to removal from the market (for metaraminol) [23] or other concerns, including compromised uterine blood flow, adverse effect on fetal acid-base status, or inconvenient preparation requirements [24]. Ephedrine's properties, including slow onset, reactive hypertension, tachyphylaxis, high placental transfer, and stimulation of fetal metabolism and potential fetal acidemia, collectively render it an inferior choice compared to PE [25].

Thus, PE is at present the first line vasopressor in obstetric spinal anesthesia [25], and its superiority in this context has been comprehensively explored with regard to different dosing regimens and maintenance mode, with reviews available [26, 27]. As a kind of sympathomimetic amine, PE is a pure *α*-adrenergic receptor agonist with no *β*-adrenergic receptor activity. It induces arteriolar vasoconstriction to increase SVR and mean arterial pressure (MAP), reflexively leading to a dose-dependent decrease of HR and, in turn, a decrease of CO [28]. In venous capacitance vessels, such vasoconstriction may increase venous return; however, venous resistance also increases, thus limiting venous return to the heart [29]. In comparison, NE has weak *β*-receptor agonist activity as well as *α*-adrenergic receptor agonism. Theoretically, NE is less likely to decrease HR and CO, rendering it a promising alternative to PE in obstetric anesthesia. Pharmacology of both drugs is summarized in Table 4 using data from multiple sources [30, 31], but these data still need to be confirmed in obstetric spinal parturients. Of note, the peak pressor time of bolus PE is 61.8 s [22] while for NE, most literature suggests an onset time of < 60 s [30–32]. Both NE and PE are catecholamines that do not readily cross the placenta. In Ngan Kee et al.' [33], a median umbilical venous to maternal arterial plasma concentration ratio is 17% for PE. For NE, clinical data are not available, but in vitro, the transfer from the maternal to the fetal side is 11.6 ± 0.6% in a perfused human placental lobe [34].

Consistent with its pharmacological properties, NE provides a maternal HR advantage compared to PE in most trials. Further, as recommended by a recent consensus statement, monitoring of maternal HR can be used as a surrogate for CO if the latter is not monitored [35]. Better maintenance of HR and a potentially greater CO is considered a significant benefit of NE. NE is considered an appropriate vasopressor in women with low baseline HR or compromised cardiac function [2]. As current evidence still suggest PE infusion is a default ‘good' approach, whether the slight HR or possible CO advantage of NE is sufficient to induce practitioners to switch to the more ‘perfect' NE is unclear [13]. More evidence might be needed to prove the benefits of NE compared over PE to motivate practitioners to make a change.

Although efficacy and safety of NE are suggested by these reports, the optimal dosing and administration paradigm is still debated. Either computer-controlled closed loop feedback infusion or manually controlled variable rate infusion of NE provides a more precise blood pressure management compared to equipotent PE infusion or NE bolus. However, such infusion regimens require smart pumps or more physician intervention that is not feasible at all institutions. In addition, a recent consensus statement notes that hypotension is frequent and, therefore, vasopressors should be used routinely and preferably prophylactically [35]. Prophylactic fixed-rate infusion is associated with less hemodynamic fluctuation and fewer maternal side effects compared to a reactive bolus. Different groups have explored the optimal fixed infusion rate for NE alone [2, 4, 7] or in comparison with PE [9], with doses ranging from 1.5 to 15 *μ*g/min (for a 60 kg parturient). Results showed that a dose 3-5 *μ*g/min is sufficient in most cases, with a higher dose (10-15 *μ*g/min) exposing parturients to hypertension.

In summary, NE is similarly effective to PE in managing maternal spinal hypotension. However, this conclusion is obtained using < 10 reports, and thus our confidence in results must remain moderate compared to the hundreds of reports available on PE efficacy and safety. Further, not all the reports in our study are high-quality ones. For example, the study of Vallejo et al.' [13] is highly questioned by a subsequent editorial. Firstly, the study design was not blinded, a serious limitation in a clinical trial. Second, a fixed infusion of PE at a dose of 6 *μ*g/min is significantly lower than the commonly used dosing range of 25-100 *μ*g/min for parturients with elective cesarean delivery and almost certainly less potent than NE 3 *μ*g/min. Such dosing bias inevitably made it difficult to detect true differences in drug efficacy or adverse outcomes. Third, continuous BP monitoring with Nexfin finger cuff made the observer more likely to intervene with hemodynamic management, potentially leading to an investigator bias. These limitations, combined with the lack of fetal umbilical artery blood gas analysis, reduce our confidence in the results of the study. Thus, based on the limited available literature, NE is likely a promising alternative to PE, but before routine application, more favorable high-quality studies are warranted.

## 5. Conclusion

Although this minireview suggests NE is effective and safe in obstetric spinal anesthesia, several uncertainties require exploration. First, CO superiority is demonstrated by the available literature for NE compared to PE, but it does not manifest any clinical advantages because no differences in maternal side effects, neonatal Apgar scoring, or blood gas analysis are observed. This may be because all enrolled parturients were healthy and without comorbidity. CO advantage should be further clarified in high risk situations, such as maternal cardiac disease, placental insufficiency, or poor fetal status. Second, certain details regarding NE application need to be worked out, including target blood pressure, infusion administration or bolus regimen, and, importantly, the optimum dose [35]. Finally, data regarding the application of NE in special circumstances is lacking, such as in women with pre-eclampsia, in women who are morbidly obese, or in institutions where resources are constrained.

The available literature suggests NE is likely a promising alternative for rescuing maternal hypotension in obstetric anesthesia. However, due to the relatively small number of available studies, it is too early to draw a definite conclusion. For assurance of routine use in obstetric anesthesia, acquisition of more high-quality supporting data of NE is warranted.

## Figures and Tables

**Table 1:** Characteristics of enrolled studies.

Reference	Study design	Participants	Anesthesia	Dosing regimen	Intervention	Observational-end point	Primary outcome variables
Hasanin et al., 2018 [2]	Double-blind RCT	284 healthy women	Spinal anesthesia with bupivacaine 10 mg + fentanyl 20 *μ*g, rapid crystalloid coloading to 1500 ml starting at subarachnoid block, left tilt supine position	Fixed rate infusion	NE infusion 0.025, 0.05, or 0.075 *μ*g/kg/min for SBP maintenance after an initial bolus of NE 5 *μ*g given post spinal anesthesia, ephedrine 9 mg is given when SBP < 80% baseline or SBP < 80% baseline + bradycardia (HR<55 bpm), ephedrine 15 mg is given for severe hypotension (SBP < 60% baseline), atropine 0.5mg for bradycardia persisted after the previous measure	Immediately post spinal anesthesia until 5 min post delivery	Frequency of post-spinal hypotension (SBP < 80% baseline)

Sharkey et al., 2018 [10]	Double-blind RCT	112 healthy women	Spinal anesthesia with bupivacaine 13.5 mg + fentanyl 10 *μ*g+ morphine 100 *μ*g, rapid hydration for 10ml/kg immediately preceding intrathecal injection, left tilt supine position	Intermittent bolus	NE bolus 6 *μ*g vs. PE 100 *μ*g whenever SBP lower than baseline, ephedrine 10 mg is given when SBP < 80% baseline + HR < 60 bpm or SBP < 80% baseline for 2 consecutive readings	Immediately post spinal anesthesia until delivery	Incidence of bradycardia (HR < 50 bpm) in the predelivery period

Ngan Kee et al., 2018 [3]	Double-blind RCT	107 healthy women	Spinal anesthesia with bupivacaine 11 mg + fentanyl 15 *μ*g, rapid hydration for 2 L post spinal anesthesia, left tilt supine position, suprasternal Doppler (USCOM) for hemodynamic monitor	Manually controlled variable rate infusion *vs. *intermittent bolus	NE infusion 0-5 *μ*g/min for SBP near baseline vs. NE bolus 5 *μ*g whenever SBP < 80% baseline	Immediately post spinal anesthesia until delivery	Incidence of hypotension (SBP < 80% baseline), and overall stability of SBP control compared with performance error (MDAPE, MDPE, Wobble)

Chen D et al., 2018 [4]	Double-blind RCT	117 healthy women	Spinal anesthesia with ropivacaine 11-12.5 mg + morphine 0.1 mg, preloading with LR 10 ml/kg, left tilt supine position, noninvasive monitoring (LIDCO) for CO and SVR	Fixed rate infusion	NE infusion 5, 10, or 15 *μ*g/kg/h *vs.* saline for SBP maintenance, NE rescue bolus 10 *μ*g for SBP < 80% baseline or < 90 mmHg	Immediately post spinal anesthesia until the end of surgery	Proportion of hypotension participants (SBP < 80% baseline or < 90mmHg)

Vallejo M et al., 2017 [7]	Open label RCT	81 healthy women	Spinal anesthesia with bupivacaine 12-15 mg + fentanyl 20 *μ*g + morphine 0.2 mg, preloading with LR 500 ml, left tilt supine position, noninvasive monitoring (Nexfin) for CO, CI, SV, SVR	Fixed rate infusion	NE 0.05 vs. PE 0.1 *μ*g/kg/min for SBP with 100-120% of baseline, rescue bolus PE 100 *μ*g for hypotension (SBP < baseline) or rescue ephedrine 5 mg for hypotension + bradycardia (HR < 60 bpm)	Immediately post spinal anesthesia until the patient care transferred to the labor and delivery nurse postoperatively	Number and type of rescue bolus intervention needed to maintain SBP

Onwochei et al., 2017 [5]	Double-blind up-down sequential allocation dose-finding study	40 pregnant women	Spinal anesthesia with bupivacaine 13.5 mg + fentanyl 10 *μ*g + morphine 0.1 mg, rapid hydration for 10ml/kg immediately preceding intrathecal injection, left tilt supine position	Intermittent bolus	NE bolus 3, 4, 5, 6, 7, or 8 *μ*g whenever SBP < 100% baseline	Immediately post spinal anesthesia until delivery	Success of NE regimen to maintain SBP at or above 80% of baseline

Ngan Kee et al., 2017 [6]	Random allocation-graded dose-response study	180 healthy women	Spinal anesthesia with bupivacaine 11 mg + fentanyl 15 *μ*g, rapid cohydration with Plasma-Lyte-A 2L, left tilt supine position	Intermittent bolus	NE bolus 4, 5, 6, 8, 10, 12 *μ*g vs. PE 60, 80, 100, 120, 160, 200 *μ*g for first episode of hypotension (SBP < 80% baseline)	Post spinal anesthesia until completion of each response measurement	Dose response curve

Ngan Kee et al., 2017 [8]	Two-arm parallel, double-blind RCT	101 healthy women	Spinal anesthesia with bupivacaine 11 mg + fentanyl 15 *μ*g, rapid cohydration with LR to maximal 2L just after intrathecal injection, left tilt supine position	Closed-loop feedback computer-controlled infusion	NE 0-5 *μ*g/min vs. PE 0-100 *μ*g/min for SBP near baseline with computer designed algorithm	Immediately post spinal anesthesia until delivery	MDAPE)

Ngan Kee et al., 2015 [9]	Two-arm parallel, double-blind RCT	101 healthy women	Spinal anesthesia with bupivacaine 11 mg + fentanyl 15 *μ*g, rapid cohydration with LR to maximal 2 L just after intrathecal injection, left tilt supine position, suprasternal Doppler (USCOM) for CO monitor	Closed-loop feedback computer-controlled infusion	NE 0-5 *μ*g/min vs. PE 0-100 *μ*g/min for SBP near baseline with computer designed algorithm	Immediately post spinal anesthesia until delivery	CO

RCT: randomized controlled trial; NE: norepinephrine; PE: phenylephrine; SBP: systolic blood pressure; HR: heart rate; LR: lactated ringers' solution; CO: cardiac output; CI: cardiac index; SV: stroke volume; SVR: systemic vascular resistance; MDAPE: median absolute performance error; MDPE: median performance error; Wobble: a measure of the variability of performance error around MDPE for each patient.

**Table 2:** Efficacy evaluation of norepinephrine.

Reference	Participants and dosing regimen	BP	HR	Non-invasive hemodynamic	Drug consumption	Performance error
Hasanin et al., 2018 [2]	Group NE1 (n=95), 0.025 *μ*g/kg/min; NE2 (n=93), 0.05 *μ*g/kg/min; NE3 (n=96), 0.75 *μ*g/kg/min; followed with an initial bolus of NE 5 *μ*g given post spinal anesthesia	SBP was higher with NE2/NE3 compared to NE1^∗^ Frequency of post-spinal hypotension: NE1 > NE2/NE3, 42.1%, 24.7%, and 26.0%, respectively^∗^ Frequency of severe hypotension, intraoperative hypertension, and postdelivery hypotension: ns	HR was lower in NE2/NE3 compared to NE1^∗^ Frequency of bradycardia requiring atropine, ns	N/A	Ephedrine requirements: NE1 > NE2/NE3, 7 ± 10, 5 ± 9, and 5 ± 9 mg, respectively^∗^	N/A

Sharkey et al., 2018 [10]	Group NE (n=56) and group PE (n=56), NE bolus 6 *μ*g vs. PE 100 *μ*g whenever SBP lower than baseline, ephedrine 10 mg is given when SBP < 80% baseline + HR < 60bpm or SBP < 80% baseline for 2 consecutive readings	Incidence of hypotension, hypertension, and tachycardia: ns Need for NE and PE bolus: ns Need for rescue ephedrine bolus: 7.2% vs. 21.4%^∗^	Incidence of bradycardia: group NE < group PE, 6 (10.9%) vs 21 (37.5%)^∗^ Incidence of > 2 episodes of bradycardia: group NE < group PE, 2(3.6%) vs 11(19.6%)^∗^	N/A	Proportion of need for ephedrine: group NE < group PE, 7.2% vs. 21.4%^∗^ Vasopressor bolus required: 9 [6-14] for NE vs. 8 [5.5-10.5] for PE, ns	N/A

Ngan Kee et al., 2018 [3]	Group NE1 (n=53), manually controlled variable rate infusion, 0-5 *μ*g/min for SBP near baseline Group NE2 (n=54), bolus 5 *μ*g whenever SBP < 80% baseline	Incidence of hypotension: group NE1 < NE2, 9 (17%) vs 35 (66%)^∗^ AUC of SBP over time: group NE1 > NE2, 110.9 ± 8.3 vs 101.9 ± 9.4 mmHg^∗^	Incidence of bradycardia: 4 (7.5%) vs 4 (7.4%), ns AUC of HR over time: group NE1 < NE2, 82.2 ± 10.4 vs 88.2 ± 12.1 mmHg^∗^	CO: 6.85 ± 1.37 for group NE1 vs 6.42 ± 1.31 L/min for group NE2, ns	Total dose: group NE1 > NE2, 61.0 (47.0-72.5) vs 5.0 (0-18.1) *μ*g Median rate: group NE1 > NE2, 2.22 (1.87-2.57) vs 0.28 (1.87-2.57) *μ*g/min	MDPE: group NE1 < NE2, -2.99 (-6.36 to 0.29) vs -11.15 (-14.77 to -7.65)^∗^ MDAPE: group NE1 < NE2, 4.97 (3.81 to 6.74) vs 11.33 (7.86 to 14.99)^∗^ Wobble, 3.13 (2.51 to 3.76) vs 3.32 (2.45 to 5.00), ns

Chen D et al., 2018 [4]	Group NE1 (n=29), 5 *μ*g/kg/h; NE2 (n=30), 10 *μ*g/kg/h; NE3 (n=28), 15 *μ*g/kg/h; control group (n=30), saline infusion; rescue bolus 10 *μ*g for SBP < 80 % baseline or < 90 mmHg	Incidence of hypotension: control group > NE1, NE2, NE3, 86.7, 37.9, 20, 25%, respectively^∗^ Incidence of hypertension: control group < NE1/NE2 < NE3, 10, 41.4, 36.6, and 75%, respectively^∗^	Incidence of bradycardia: 0, 3.4, 3.3, 10.7 %, ns HR: ns among groups	CO, SVR: ns	Total dose: control group < NE1 < NE2, NE3, 23 ± 20, 186.9 ± 79.6, 375.8 ± 137.3, 479.1 ± 243.8*μ*g, respectively^∗^	N/A

Vallejo M et al., 2017 [7]	Group NE (n=43), 0.05 *μ*g/kg/min; group PE (n=38), 0.1 *μ*g/kg/min; for SBP with 100-120% of baseline, rescue bolus PE 100 *μ*g for hypotension (SBP < baseline) or rescue ephedrine 5 mg for hypotension + bradycardia (HR < 60 bpm)	Proportion of vasopressor requirement: 21 (48.8%) for group NE and 25 (65.8%) for group PE, ns Requirement for PE rescue bolus: 20 (46.5%) for group NE and 20 (52.6%) for group PE, ns Requirement for ephedrine rescue bolus: group NE < PE, 1 (2.3%), 9 (23.7%)^∗^ Incidence of hypertension: 1 (2.6%) vs 2 (4.7%), ns	Incidence of bradycardia: 8 (18.6%) for group NE and 9 (23.7%) for group PE, ns	CO, CI, SV, SVR: ns	Median total rescue PE dose: 100 [0-700] for group NE and 50 [0-1000]*μ*g for group PE, ns Median total rescue ephedrine dose: 0 [0-30] for group NE and 0 [0-85] mg for group PE, ns	N/A

Onwochei et al., 2017 [5]	Bolus NE 3 (n=6), 4 (n=2), 5 (n=9), 6 (n=20), 7 *μ*g (n=3) to rescue first episode of hypotension	ED90 of NE 5.49 *μ*g (95%CI 5.15-5.83) using truncated Dixon and Mood method; 5.80 *μ*g (95% CI 5.01-6.59) using the isotonic regression method Hypotension incidence: 6 (15%), of which 5 (83.3%) with NE < 6*μ*g; Incidence of hypertension: 4 (10%)	Incidence of bradycardia: 3 (7.3%)	N/A	Cumulative NE dose: 6 to 78 *μ*g	N/A

Ngan Kee et al., 2017 [6]	NE bolus 4, 5, 6, 8, 10, 12 *μ*g vs. PE 60, 80, 100, 120, 160, 200 *μ*g (n=15 for each dose group) for first episode of hypotension	ED50 for NE and PE is 10 *μ*g (95%CI, 6-17*μ*g), 137 *μ*g (95% CI, 79 -236*μ*g) ED90 for NE and PE is 18 *μ*g (95%CI, 5-63*μ*g), 239 *μ*g (95% CI, 66-869*μ*g) Potency ratio of NE compared to PE: 13.1 (95% CI, 10.4- 15.8)	HR decrease: NE< PE^∗^	N/A	N/A	N/A

Ngan Kee et al., 2017 [8]	NE 0-5 *μ*g/min (n=49) *vs.* PE 0-100*μ*g/min (n=52) for SBP near baseline with computer designed algorithm	Incidence of hypotension: 4(8.2%) for group NE, 4(7.7%) for group PE, ns Incidence of hypertension: 4 (8.2%) for group NE, 9 (17.3%) for group PE, ns	Incidence of bradycardia: 9 (18.4%) for group NE, 29 (55.8%) for group PE^∗^	N/A	Total vasopressor volume: 10.4 [9.5-14.1], 14.3 [9.9-16.9] ml, ns	MDPE (%): group NE < PE, 0.75 [-1.56 to 2.52] vs. 2.61 [0.83 to 4.57]^∗^ MDAPE (%): group NE < PE, 3.79 [2.82 to 5.17] vs. 4.70 [3.23 to 6.57]^∗^ Wobble (%): 2.85 [2.07 to 3.92] vs. 3.39 [2.62 to 4.90]^∗^ Divergence (% /min): ns

Ngan Kee et al., 2015 [9]	NE 0-5 *μ*g/min (n=49) *vs.* PE 0-100*μ*g/min (n=52) for SBP near baseline with computer designed algorithm	AUC of SBP over time: ns	AUC of HR over time: group NE > PE^∗^	AUC of CO: group NE > PE^∗^ AUC of SV: ns AUC of SVR: group NE < PE^∗^	N/A	N/A

NE: norepinephrine; PE: phenylephrine; MDPE: median performance error; MDAPE: median absolute performance error; SBP: systolic blood pressure; HR: heart rate; CO: cardiac output; CI: cardiac index; SV: stroke volume; SVR: systemic vascular resistance; AUC: area under the curve; Values are mean (standard deviation), median [interquartile range], or number (%);^∗^*P* <0.05; ns: no statistical significance between groups; N/A: not available.

**Table 3:** Safety evaluation of norepinephrine.

Reference	Maternal side effects	Anticholinergic drug	Apgar scoring	Blood gas analysis	Umbilical blood catecholamines
Hasanin et al., 2018 [2]	Nausea or vomiting: ns	Four in NE1, 3 in NE2, and 7 in NE3: ns	Apgar at 1 and 10 min: ns	pH, PCO_2_, PO_2_, HCO in UA: ns	N/A

Sharkey et al., 2018 [10]	Nausea or vomiting: ns	N/A	Apgar at 1 and 5 min: ns	pH, PCO_2_, PO_2_, HCO_3_, BE in UA and UV: ns	N/A

Ngan Kee et al., 2018 [3]	Nausea or vomiting: ns	No patient	No Apgar < 7 at 1min or < 8 at 5min in either group	pH, PCO_2_, PO_2_, BE in UA and UV: ns	N/A

Chen D et al., 2018, [4]	Shivering, nausea, and pale skin: ns	One in NE3: ns	Apgar at 1 and 5 min: ns	Glucose: NE2, NE3 > NE1, control group in either maternal or neonatal UA; pH, PCO_2_, PO_2_, HCO_3_, Lac, BE: ns among groups in maternal and neonatal UA	N/A

Vallejo M et al., 2017 [7]	Nausea: 51.2% for group NE and 63.2% for group PE, ns; emesis: 16.3% for group NE vs. 26.3% for group PE, ns	N/A	Apgar < 7 at 1min or 5min: ns	pH, PCO_2_, PO_2_, HCO_3_, BE in UV: ns	N/A

Onwochei et al., 2017 [5]	Nausea: 11 (27.5%), of which 4 (36.4%) and 7 (63.6%) with NE < 6 *μ*g and at 6 *μ*g, respectively; no vomiting observed	N/A	All Apgar > 8 at 1 min or 5 min	Incidence of UA pH < 7.2: 6 (15%), 1 with NE < 6*μ*g, 5 with NE 6 *μ*g BE is higher in those with NE < 6*μ*g compared to those with 6 *μ*g: -2.56 vs -4.94^∗^	N/A

Ngan Kee et al., 2017 [6]	N/A	N/A	Apgar < 7 at 1 min or < 8 at 5 min: ns	Incidence of UA pH < 7.2: 4 (4.4%) vs 5(5.6%), ns	N/A

Ngan Kee et al., 2015 [9]	Nausea or vomiting: 3 (6.1%) for group NE and 2 (3.8%) for group PE, ns	N/A	All Apgar > 7 at 1 min and 5 min	No patient had UA pH < 7.2 pH and oxygen content: group NE > PE in UV^∗^; PCO_2_, PO_2_, BE: ns in UV; pH, PCO_2_, PO_2_, BE, oxygen content, ns in UA Glucose content: group NE > PE in UA and UV^∗^	Epinephrine content: group NE < PE in UA^∗^; NE content: group NE < PE in UA and UV^∗^

BE: base excess; NE: norepinephrine; PE: phenylephrine; UA: umbilical artery; UV: umbilical vein; ns: no significant difference between or among groups; values are number (%); N/A: not available.

**Table 4:** Pharmacology of phenylephrine and norepinephrine.

	Phenylephrine	Norepinephrine
Pharmacology	*α*1, *α*2	*α*1, *α*2, *β*1>>*β*2

Onset time	60 s	< 60s

Half time	5 min	1-2 min

Molecular structure	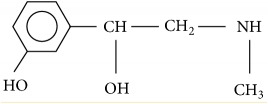	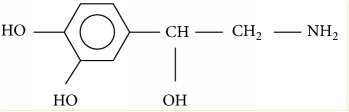

Molecular weight	167 g/mol	169 g/mol

Metabolism	Deamination, glucuronidation, sulfation	COMT+MAO to VMA

Relative potency	1×	13×

Placental transfer	Minimal	Likely minimal too

Fetal metabolism stimulation	Lower than ephedrine	Lower than phenylephrine

MAP	↑	↑

HR	Dose dependently ↓	± or ↓

SV	±	± or ↑

CO	Dose dependently ↓	± or ↑

SVR	↑	↑

Venous resistance	↑	±

Venous return	±	±

Myocardial contractility	± or ↓	↑

COMT: catechol-O-methyltransferase; MAO: monoamine oxidase; VMA: vanillylmandelic acid; MAP: mean arterial pressure; HR: heart rate; SV: stroke volume; CO: cardiac output; SVR: systemic vascular resistance; ± indicates neutral effect.
